# Whole Genomic Sequence Analysis of Human Adenovirus Species C Shows Frequent Recombination in Tianjin, China

**DOI:** 10.3390/v15041004

**Published:** 2023-04-19

**Authors:** Yue Lei, Zhichao Zhuang, Yang Liu, Zhaolin Tan, Xin Gao, Xiaoyan Li, Dongjing Yang

**Affiliations:** Tianjin Key Laboratory of Pathogenic Microbiology of Infectious Disease, Tianjin Centers for Disease Control and Prevention, Tianjin 300011, China; leiyue1983@163.com (Y.L.); dennis.eyre@hotmail.com (Z.Z.); liuyang_198002@163.com (Y.L.); tzl353355149@163.com (Z.T.); syzxgx123@163.com (X.G.)

**Keywords:** human adenovirus, whole genome sequencing, recombination analysis

## Abstract

Human adenovirus species C (HAdV-C) is frequently detected in China and worldwide. For the first time, 16 HAdV-C strains were isolated from sewage water (14 strains) and hospitalised children with diarrhoea (2 strains,) in Tianjin, China. Nearly complete genome data were successfully obtained for these viruses. Subsequently, genomic and bioinformatics analyses of the 16 HAdV-C strains were performed. A phylogenetic tree of the complete HAdV-C genome divided these strains into three types: HAdV-C1, HAdV-C2, HAdV-C5. Phylogenetic analysis based on the fiber gene showed similar outcomes to analyses of the hexon gene and complete HAdV-C genomes, whereas the penton gene sequences showed more variation than previously reported. Furthermore, analysis of the whole-genome sequencing revealed seven recombination patterns transmitted in Tianjin, of which at least four patterns have not been previously reported. However, the penton base gene sequences of the HAdV-C species had significantly lower heterogeneity than those of the hexon and fiber gene sequences of recombinant isolates; that is, many strains were distinct in origin, but shared hexon and fiber genes. These data illustrate the importance of frequent recombination in the complexity of the HAdV-C epidemic in Tianjin, thus emphasising the necessity for HAdV-C sewage and virological monitoring in China.

## 1. Introduction

Human adenovirus (HAdV) is a non-enveloped, linear, double-stranded DNA virus with icosahedral symmetry and a genome size of approximately 34–36 kb [1]. To date, more than 113 human HAdV types have been identified and classified into seven groups (HAdV-A to -G), with 56 serotypes recognised by the Human Adenovirus Working Group as of March 2022 “http://hadvwg.gmu.edu/ (accessed on 1 December 2022)”. The original classification of serotypes 1–51 was performed through serum neutralisation assays and haemagglutination inhibition tests, and serotypes from 52 onwards are now continuously updated based on genomic and bioinformatics data [1,2].

Although human adenovirus species C (HAdV-C) infections are common, only eight types have been identified (HAdV-C1, C2, C5, C6, C57, C89, C104 and C108) (http://hadvwg.gmu.edu/ (accessed on 1 December 2022) [3]. HAdV-C1 and HAdV-C2 are responsible for more than half of adenovirus infections in immunocompromised individuals. HAdV-C57 was identified in a stool sample from a healthy child during a surveillance program for acute flaccid paralysis in 2001 [4]. Two HAdV-C89 strains were isolated from different patients in 2015 and 2017 [5] and HAdV-C104 was isolated from a respiratory specimen of a child in China in 2017 [6]. Meanwhile, HAdV-C108 was published in 2014, but without any reference strains “http://hadvwg.gmu.edu/ (accessed on 1 December 2022)”. After the initial infection, HAdV-C may remain latent in lymphoid cells asymptomatically and shed an infectious virus intermittently in faeces for extended periods [7]. The double-stranded DNA structure of adenovirus makes it more thermally stable and provides a longer lifespan in water than that of the enterovirus [8]. Additionally, its capacity to use host cells to fix damaged DNA allows it to remain in the environment for an extended period [9].

Homologous recombination is the main driver of HAdV molecular evolution and variation, especially the recombination between the hexon, penton base, and fiber genes, which has been confirmed in HAdV-B and HAdV-D, but is not common in HAdV-C [10]. The hexon gene and genomic region flanking the fiber gene are two potential sites for HAdV-C recombination [5]. The strains HAdV-C57(P1H57F6), HAdV-C89(P89H2F2), HAdV-C104(P1H1F2), and HAdV-C108(P1H2F2) have all been categorized as recombinants “http://hadvwg.gmu.edu/ (accessed on 3 December 2022)” [4,5]. The current research identified two HAdV-C strains from faecal samples of hospitalised children under five years of age in Tianjin, in addition to 14 adenovirus strains from sewage. The penton, hexon, and fiber genes of these 16 HAdV-C strains were identified, and whole genome sequences of the viruses were generated for recombination analysis. These results suggest that a series of recombinant HAdV-C strains may circulate in the human population in Tianjin, China.

## 2. Materials and Methods

### 2.1. Sample Collection and Processing

Sewage samples were collected from the Zhang GZ wastewater treatment plant (WWTP) located in Tianjin, China, from 2021 to March 2022. Each month, a 5 L sample was obtained from the inlets of the WWTP. The samples were then immediately transported to the laboratory. The negative-charge membrane adsorption ultrasonic elution method was used to concentrate 1 L of each sewage sample into a 10 mL eluent [11]. The Tianjin adenovirus strains (TJ-149-2022, TJ-165-2021) were isolated from inpatients that were one and three years old, respectively, and had been diagnosed with diarrhoea at Tianjin Children’s Hospital.

### 2.2. Virus Isolation

Human laryngeal carcinoma epithelial cells (HEp-2) were inoculated with 200 µL eluent in Dulbecco’s modified Eagle’s medium containing 2% foetal bovine serum. After incubation at 37 °C for seven days, if no cytopathic effect (CPE) was observed, the culture supernatants were used for two additional passages. If adenovirus-like CPE was observed, the cultures were passaged again to confirm the presence of the virus in high-titre stocks. Virus-incubated cells and supernatants were collected and subjected to genome sequencing.

### 2.3. DNA Extraction and Next-Generation Sequencing

A QIAamp MinElute virus spin kit (Qiagen, Hilden, Germany, Cat. No. 52904) was used to extract viral genomic DNA, according to the manufacturer’s instructions. A polymerase chain reaction was performed using the extracted viral DNA as a template, adenovirus universal primers, and the PrimeSTAR Max DNA Polymerase (TaKaRa R045A, Shiga, Kusatsu, Japan) kit, according to the manufacturer’s instructions. Positive samples were sent to the Shanghai BioGerm Medical Technology Co., Ltd. (Shanghai, China) for sequencing. The sequencing data were compared and analysed using BLASTN in the GenBank database, and the samples were confirmed to be HAdV-C. An ULSEN ultra sensitive adenovirus whole genome capture kit (Group C) (B-170931, Beijing Micro Future, Beijing, China) was used to amplify the viral genome. An Illumina Nextera XT DNA library preparation kit (FC-131-1096, Illumina, San Diego, CA, USA) was used to construct a next-generation sequencing library, and a MiniSeq sequencer (Illumina, San Diego, CA, USA) was used for sequencing. The experiments were conducted according to the manufacturer’s instructions provided in the kit.

### 2.4. Phylogenetic Analysis

A CLC Genomics Workbench 22.0 (Qiagen, Germany) was used to process the off-machine sequencing data. The entire genome sequence of the virus was assembled using the whole genome sequence of the adenovirus standard strain (NC_001405) in the National Center for Biotechnology Information database as a template. Multiple Alignment using Fast Fourier Transform software was employed for multiple sequence alignment, and the neighbour-joining method in MEGA 7.0 software was implemented to construct phylogenetic trees based on the entire genome, as well as the hexon, penton base, and fiber genes [12] “https://mafft.cbrc.jp/alignment/software/ (accessed on 5 January 2023)”. The Kimura-2 nucleoside acid substitution model (Kimura-2-parameter model) was chosen to evaluate the credibility of the results, using a bootstrap value of 1000. Based on the whole-genome sequences (WGSs), the phylogenetic network was generated using a SplitsTree4 software version 4.14.6 “http://www.splitstree.org/ (accessed on 5 January 2023)”.

### 2.5. Recombination Analysis

Recombination detection program (RDP) version 4.97 software was employed to analyse potential recombination events among the sequences, using the seven algorithms (RDP, GENECONV, Bootscan, Maxchi, Chimaera, SiSscan, and 3Seq) provided by the software with the parameters set to default values [13]. SimPlot version 3.5.1 software was then used to assess the reliability of the RDP recombination results, with the parameters set to the Kimura-2-parameter model, the nucleotide conversion and transversion rate ratio set to 2.0, the window set to 200, and the step set to 20 base pairs (bp) https://sray.med.som.jhmi.edu/SCRoftware/simplot/ (accessed on 7 January 2023).

## 3. Results

### 3.1. Full-Length Genomic Characterisation

The 16 adenovirus strains were confirmed by a visible CPE in HEp-2 cells. The strains were further characterised using next-generation sequencing. The 16 WGSs span a length of 35,390–35,900 bp and the average GC content is 55.27%, which is typical of HAdV-C genomes, as shown in Table 1.

When we conducted pairwise comparisons of the six prototype strains of HAdV-C1 (AC000017), HAdV-C2 (AC000007), HAdV-C5 (AC000008), HAdV-C6 (HQ413315), HAdV-C57 (HQ003817), and HAdV-C89 (MH121097), we found that TJ-ET149-2022 and three TJ-Sewage strains had the greatest similarity to HAdV-C1 (99.03–99.31%), TJ-ET165-2021 and seven TJ-Sewage strains had the greatest similarity to HAdV-C2 (98.94–99.68%), while three TJ-Sewage strains had the greatest similarity to HAdV-C5 (98.22–99.00%), and only one TJ-Sewage strain (21110206) had similarity to HAdV-C89 (99.52%) (Table 2).

When we compared the 16 adenovirus sequences with those in the GenBank database, we found that the sequences of TJ-ET149-2022, TJ-Sewage-210202, and TJ-Sewage-210402 showed the highest similarity (99.77%) to MT263140 (LN2017, isolated from a faecal specimen of a patient with acute flaccid paralysis in Liaoning Province, China in 2017). However, the sequence similarity with the prototype strain HAdV-1 was only 99.03%. TJ-ET165-2021, TJ-Sewage-210406, TJ-Sewage-21120204, and TJ-Sewage-22020104 showed the greatest similarity to ON054624 (HK61/P1H2F2), ranging from 99.82% to 99.90%. However, the sequence identity with the prototype strain HAdV-2 was only 99.03–99.08%. TJ-Sewage-21,110,103 and 22,030,101 showed a greater similarity (99.88%) to MF315029 (BJ09, a strain isolated from a patient with a respiratory infection in Beijing, China, in 2013) than the prototype strain of HAdV-2 (98.94%) (Table 3).

### 3.2. Phylogenetic Analysis

**Figure 1:**
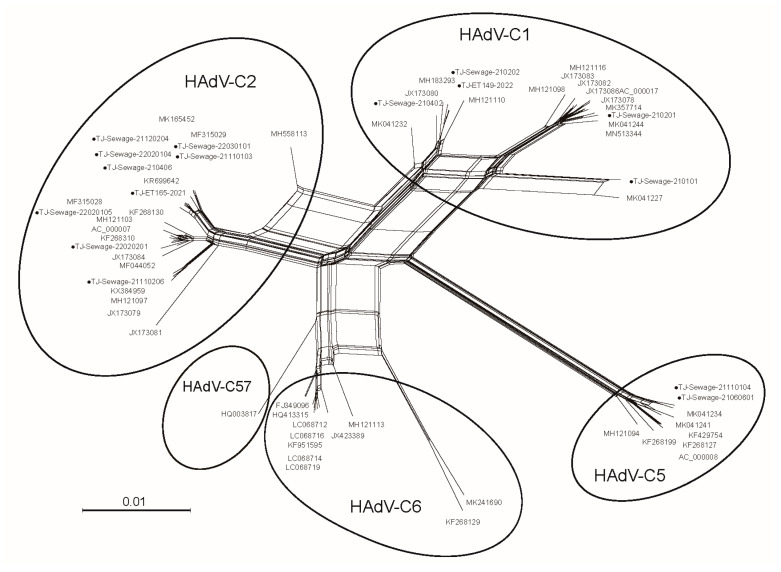
Phylogenetic network built using the complete genomes of 16 Tianjin human adenovirus (HAdV) (represented by black dots) and 52 human adenovirus species C (HAdV-C) strains obtained from the GenBank database.

A phylogenetic network was generated based on the genomes of 16 Tianjin HAdV and 52 HAdV-C strains obtained from the GenBank database. There were at least four major clusters representing genotypes C1, C2, C5, and C6. However, sub-clusters were obvious in clusters C1, C2, and C6, indicating different evolutionary pathways (Figure 1).

**Figure 2:**
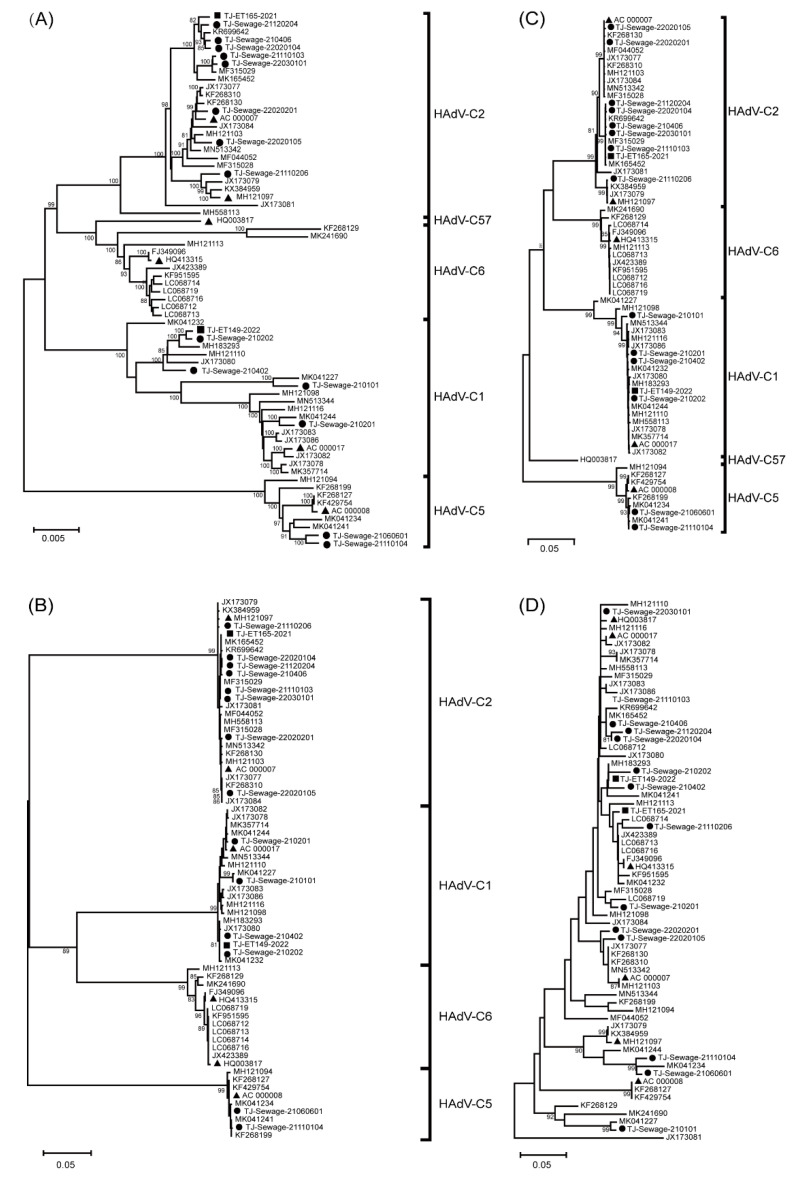
Phylogenetic trees based on the complete genome (**A**), fiber gene (**B**), hexon gene (**C**) and penton gene (**D**) of 68 HAdV-C sequences, including 16 Tianjin strains in this study and 52 reference strains from the GenBank. Tianjin sequences isolated from faeces are indicated with a black square, whereas sequences isolated from sewage are indicated with a black dot. Each prototype sequence is indicated with a black triangle. The trees were constructed using the Neighbour-joining method of MEGA 7.0 with 1000 bootstraps. HAdV-C, human adenovirus species C.

Phylogenetic analysis was also performed to determine the genetic relationships among the 16 Tianjin and 52 HAdV-C strains acquired from the GenBank database. Based on the phylogenetic tree of complete HAdV-C genomes, the 14 strains isolated from sewage were divided into three types: HAdV-C1, HAdV-C2, and HAdV-C5, while the two strains isolated from patients were more similar to HAdV-C1 and HAdV-C2. More specifically, in the HAdV-C2 clade, five sewage strains (TJ-Sewage-21120204, TJ-Sewage-210406, TJ-Sewage-22020104, TJ-Sewage-21110103, and TJ-Sewage-22030101) and the TJ-ET165-2021 cluster with KR699642 and MF315029, which have been reported as recombinant HAdV-C2 strains in previous studies [14]. TJ-Sewage-22020201 and TJ-Sewage-22020105 were closely related to the prototype HAdV-C2 strain (AC000007), whereas TJ-Sewage-21110206 was more closely related to the prototype HAdV-C87 strain (MH121097). Similarly, in the HAdV-C1 clade, TJ-ET149-2022, TJ-Sewage-210202, and TJ-Sewage-210402 formed a subclade with MH183293, MH121110, and JX173080, whereas TJ-Sewage-210101 clustered with MK041227, forming another unique subclade with relatively long branch lengths within the HAdV-C1 clade. Furthermore, TJ-Sewage-21060601 and TJ-Sewage-211101104 clustered with the recombinant strain MK041241 within the HAdV-C5 clade (Figure 2A).

To classify the 16 strains, the penton base, hexon, and fiber genes were analysed separately. Phylogenetic analysis based on the fiber gene showed similar results to those for the hexon gene, with 16 strains belonging to types 1, 2, and 5 (Figure 2B). According to the phylogenetic analysis based on the hexon gene, the 16 Tianjin strains were clearly divided into three types: 1, 2, and 5. TJ-Sewage-21120204, TJ-Sewage-210406, TJ-Sewage-22020104, TJ-Sewage-21110103, TJ-Sewage-22030101, and TJ-ET165-2021 were classified as type 2. TJ-Sewage-210101, TJ-Sewage-210201, TJ-Sewage-210202, TJ-Sewage-210402, and TJ-ET149-2022 were classified as type 1, whereas TJ-Sewage-21060601 and TJ-Sewage-21110104 were classified as type 5 (Figure 2C). However, phylogenetic analysis based on the penton base gene yielded different results. Nine strains, including five HAdV-C2 strains (TJ-Sewage-21120204, TJ-Sewage-210406, TJ-Sewage-22020104, TJ-Sewage-21110103, and TJ-Sewage-22030101) and four HAdV-C1 strains (TJ-ET149-2022, TJ-Sewage-210202, TJ-Sewage-210402, and TJ-Sewage-210201), were identified as type 1, whereas two HAdV-C2 strains (TJ-ET165-2021 and TJ-Sewage-21110206) and one HAdV-C1 strain (TJ-Sewage-210101) showed penton base gene sequence clustering with type 6. Furthermore, two HAdV-C5 strains (TJ-Sewage-21110104 and TJ-Sewage-21060601) were more closely related to type 89 with respect to the penton base gene sequences (Figure 2D).

### 3.3. Recombination Analysis

**Figure 3:**
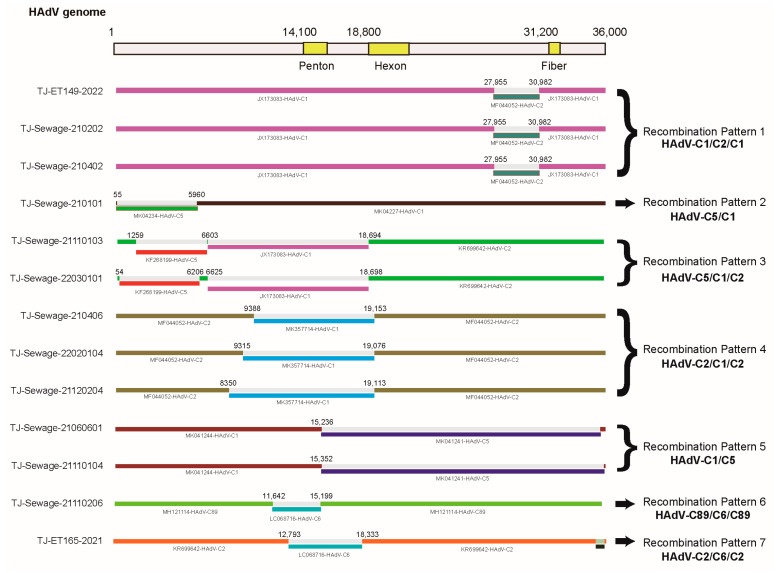
Schematic representation of recombination events in 13 genomes within 7 recombination patterns. A genetic map of human adenovirus species C (HAdV-C) is shown at the top. Analysed genomes are represented by a blue rectangle, the major parents are represented by a green rectangle, while the minor parents are represented by a purple rectangle. Breakpoints are identified based on the recombination detection program (RPD) version 4 output.

**Figure 4:**
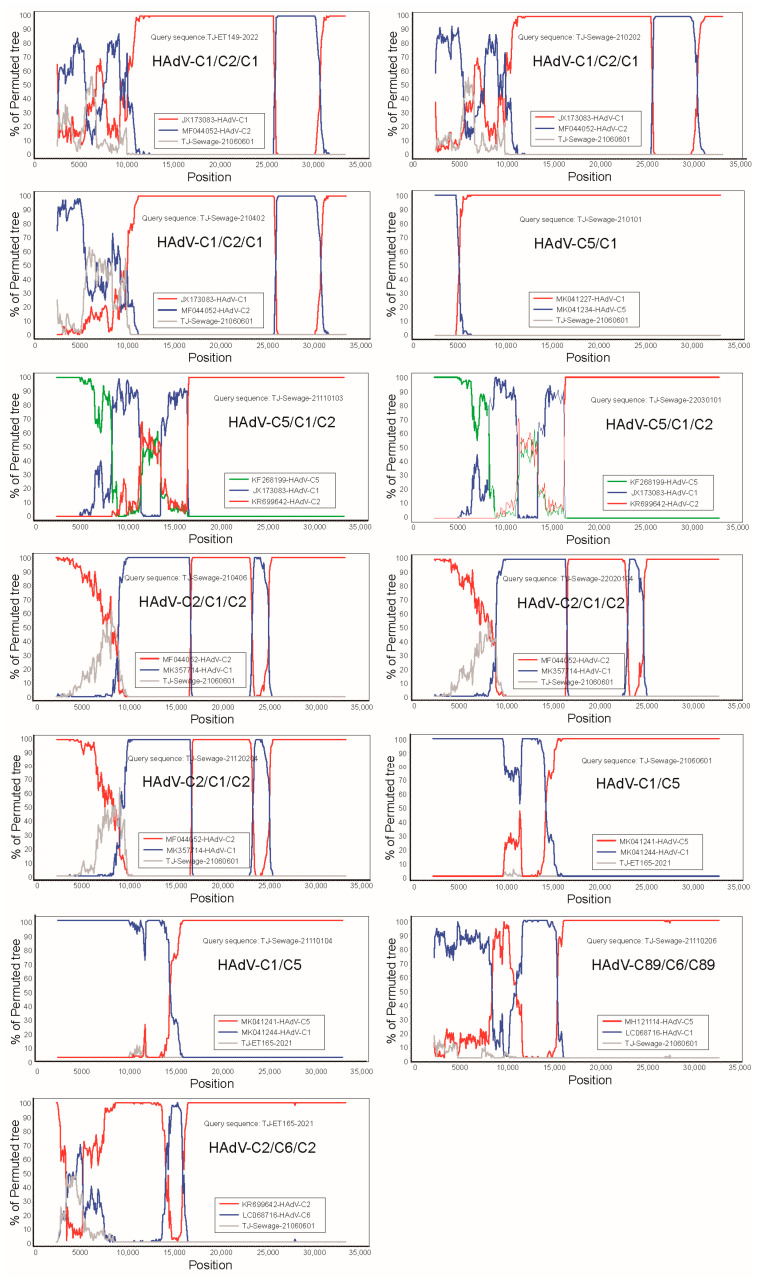
Bootscanning analysis of the 13 Tianjin HAdV-C genomes using a sliding window of 200 nt moving in 20-nt steps. For each bootscanning analysis, the names of viruses of the query sequence were indicated in the plot.

To investigate the possibility of recombination events, RDP version 4.97 and SimPlot version 3.5.1 software were used to analyse the 13 Tianjin HAdV-C strains and a total of seven distinct recombination patterns was identified (Figure 3 and Figure 4).

The first recombination pattern revealed that TJ-ET149-2022, TJ-Sewage-210202, and TJ-Sewage-200402 were products of recombination of the major parent HAdV-C1 (JX173083-USA-2003) and the minor parent HAdV-C2 (MF044052-CHN-2014). The starting point of this recombination was located at position 28,045 of HAdV-C1 (within gp12.5 kD of the E3 gene) and the end point was situated at position 31,042 of HAdV-C1, encompassing the genes encoding the E3 and fibre proteins. The RDP version 4 (RDP4) software package containing seven algorithms (RDP, GENECONV, BootScan, MaxChi, Chimaera, SiScan, and 3Seq) was used to predict potential recombination events, with *p* values ranging from 3.604 × 10^−183^ to 4.419 × 10^−19^. Simplot software confirmed the recombination events within the three strains.

The second recombination pattern, TJ-Sewage-210101, was likely a product of homologous recombination between MK041234 (HAdV-5) and MK041227 (HAdV-1), with the breakpoint beginning around the inverted terminal repeat (ITR) in early region 1A (E1A), early region 1B, protein IX, and packaging protein Iva2, and ending at position 5960 in the DNA polymerase. This event was supported by seven algorithms, with *p* values ranging from 3.914 × 10^−66^ to 2.220 × 10^−15^. SimPlot analysis revealed that the TJ-Sewage-210101 genome was composed of a mosaic structure, which was derived not only from the predominant viral strain, prototype HAdV-1, but also from HAdV-5, confirming the occurrence of recombination events.

For the third recombination pattern, BootScan and RDP4 analyses confirmed three recombination events between the TJ-Sewage-21110103 and TJ-Sewage-22030101 genomes. CBJ113 (KR699642, HAdV-2) is likely the backbone of TJ-Sewage-21110103 and TJ-Sewage-22030101, and KF268199 (HAdV-5) and JX173083 (HAdV-1) are potential genetic constituents. The breakpoint between CBJ113 and JX173083 was likely located at position 18,694 within the pre-protein VI (pVI) gene. The next breakpoint in the second recombination event between CBJ113 and KF268199 was likely located at position 6603 within the DNA polymerase gene. The end breakpoint in the third recombination event between KF268199 and CBJ113 was at position 1259 in the gene coding for the control protein E1A. SimPlot analysis of the BootScan output provided evidence of a recombination event, with *p* values ranging from 5.498 × 10^−46^ to 3.145 × 10^−4^ for 22,030,101 and 3.421 × 10^−46^ to 1.914 × 10^−5^ for TJ-Sewage-21110103.

Analysis with SimPlot software revealed that the three strains, TJ-Sewage-210406, TJ-Sewage-22020104, and TJ-Sewage-21120204, had the same recombination pattern. The fourth recombination pattern comprised the penton base gene of HAdV-1 and the hexon and fiber genes of HAdV-2. Additionally, partial sequences of DNA polymerase and E4 open reading frame 1 originated from HAdV-6 and HAdV-5, respectively. RDP analysis of the four sequences demonstrated a mosaic structure composed of gene regions originating from the two prevalent viral strains, HAdV-1 and HAdV-2. The BootScan output, supplemented by SimPlot analysis, verified the recombination event, with *p* values ranging from 1.795 × 10^−52^ to 1.465 × 10^−10^. The 21,120,204 recombinant had two breakpoints located at positions 8350 and 19,113, whereas breakpoints at 9315 and 19,076 were located in the TJ-Sewage-21120204 strain, and breakpoints at 9388 and 19,153 were located in the TJ-Sewage-210406 strain. The starting position of the recombination event in the TJ-Sewage-21120204 strain was in the DNA polymerase gene, whereas the starting position in the other two strains was in the precursor terminal protein gene, and the ending position of all three strains was in the hexon gene.

BootScan and RDP4 analyses indicated that TJ-Sewage-21060601 and TJ-Sewage-21110104 were highly likely to have undergone homologous recombination, which was attributed to AdV-1 (MK041244) and AdV-5 (MK041241). A recombinant event was identified with starting breakpoints at approximate positions 15,352 and 15,236 for the two strains and a finishing breakpoint at an approximate position of 34,917 in the 3′ ITR gene. This event included the major parent strain HAdV-1 and the minor parent strain HAdV-5, and encompassed the majority of the E4 gene. BootScan analysis was performed using SimPlot software to confirm recombination events within the genomes of TJ-Sewage-21060601 and TJ-Sewage-21110104, with *p* values ranging from 2.382 × 10^−121^ to 6.752 ×10^−13^.

TJ-Sewage-21110206 was a recombinant of the major parent MH121114 (HAdV-89) and the minor parent LC068716 (HAdV-6), with a starting breakpoint at position 11,642 in the 52k gene and an ending breakpoint at position 15,199 in the penton gene, with *p* values ranging from 1.321 × 10^−35^ to 2.595 × 10^−9^.

Six of the seven algorithms (not SiScan) supported recombination of the backbone of KR699642 (HAdV-2) and a part of LC068716 (HAdV-6) to form TJ-ET165-2021, starting at position 12,793 in the precursor protein pIIIa gene and ending at position 18,333 in the pVI gene, with *p* values from 1.754 × 10^−33^ to 5.703 × 10^−11^ (Table 4).

## 4. Discussion

For the first time, 16 adenovirus strains were isolated from sewage samples (14 strains) and from hospitalised children with diarrhoea (two strains: TJ-ET149-2022 and TJ-ET165-2021) in Tianjin, China, and the complete genome data of these viruses were successfully obtained. Analysis of the penton base, hexon, and fiber genes and whole-genome phylogenetic trees demonstrated considerable genetic diversity among the 16 HAdV-C strains. The phylogenetic tree revealed that the most common molecular types of HAdV-C in Tianjin were HAdV-1, HAdV-2 and HAdV-5. The penton base gene of HAdV-1 and the hexon and fiber genes of HAdV-2 are present in the recombinants TJ-Sewage-210406, TJ-Sewage-22020104, and TJ-Sewage-21120204, indicating that they should be categorized as HAdV-C108. Despite the absence of a reference strain on the human adenovirus working group website, the similarity between these recombinants and HAdV-C108 justifies this classification. The presence of nucleic acid diversity in the evolutionary tree and the strong similarity with typical recombinant strains implied that homologous recombination and molecular evolution analyses should be conducted on HAdV-C WGSs.

In 2019, Rivailler et al. analysed the recombination and diversity of publicly available HAdV-C genome sequences. The authors identified 20 sequences that may be used for future HAdV-C WGS recombination analysis and suggested two main breakpoint regions of HAdV-C recombination, located within the hexon gene and around the fiber genomic region [15]. Moreover, Mao et al. sequenced the genomes of 24 HAdV-C viruses circulating in mainland China from 2000–2016 and identified 16 new genetic patterns based on penton base, hexon, and fiber gene sequences, confirming that HAdV-C genomes undergo frequent recombination events [16].

Using RDP4 and Simplot software, we identified seven distinct genetic patterns and potential genotypes. Furthermore, three strains, two sewage strains (TJ-Sewage-210202 and TJ-Sewage-200402) and one strain isolated from a child (TJ-ET149-2022), were identified as recombinant HAdV-C strains. Sequence comparisons revealed that the three Tianjin adenovirus strains, together with Egyptian strain E13 isolated in 2001, Shanghai strain SH2016 isolated in 2016, and German strain 43C1, share a similar recombination pattern, beginning with the E3 gene and ending with the fibre gene [5,17]. Dhingra et al. reveal that the E3 gene of HAdV-C is highly conserved, and E3 genes of different types are remarkably distinct and have co-evolved with the hexon and fibre genes [5]. This recombination event involves the major parent HAdV-1 (JX173083-USA-2003) and minor parent HAdV-2 (MF044052-CHN-2014). The long period of isolation of the virus strains and their diverse geographical sources demonstrated the stability of this recombination model, indicating its wide prevalence and extended duration. This likely represents a novel subtype of HAdV-1.

The results of the evolutionary tree and gene recombination analysis of TJ-Sewage-210406, TJ-Sewage-21120204, and TJ-Sewage-22020104 demonstrate that these four adenoviruses are related to CBJ113, a virus collected in Beijing in 2009, and share the same recombination pattern [18]. This recombination pattern suggested two likely homologous recombination events, which were possibly derived from the parent strains HAdV-2 (MF044052-CHN-2014) and HAdV-1 (MK357714-DT-2017). However, TJ-Sewage-21110103 and TJ-Sewage-22030101 shared the same genetic restructuring as BJ09, comprising genetic elements from HAdV-1 (JX173083-USA-2003), HAdV-5 (KF268199-USA-2008), and CBJ113. Strains BJ09 and CBJ113 belong to a domestic lineage, from which a recombinant strain has been derived and subsequently discovered in sewage and human samples [19]. This suggested that CBJ113-like strains have been widely circulating in China and may have become stable epidemic strains.

To our knowledge, this is the first detection of the same recombinant strain of HAdV-C from sewage and humans in China, and its full sequence analysis has verified its presence in the environment and its stable occurrence in humans. Although the presence of HAdV-C strains in wastewater and stool samples does not necessarily link the strains with diarrhoeal symptoms, HAdV continues to be shed in faeces, even after the virus has been present in other organs [20]. After recombination analysis, two new recombination patterns were identified in sewage isolates, which yielded interesting conclusions.

TJ-Sewage-21060601, TJ-Sewage-21110104, and TJ-Sewage-210101 were all engaged in the reorganisation of HAdV-1 and HAdV-5; however, they were categorised into two different recombination patterns, a result that has not been previously reported. TJ-Sewage-21060601 and TJ-Sewage-21110104 were the products of the parent strains, Shanxi-2000-105 (HAdV-5, MK041241) and Shanxi-2006-32 (HAdV-1, MK041244). The progenitors of TJ-Sewage-210101 were Shanxi-2002-22 (HAdV-5, MK041234) and Shanxi-2010-106 (HAdV-1, MK041227). Interestingly, these four parent strains all originate from Shanxi, the same province that is the source of four new recombination patterns that are prevalent on the Chinese mainland, as noted by Mao et al. [16]. Three of the parent strains originate from the recombination of previously reported genomes; however, Shanxi-2010-106 may be the product of genome recombination, which is yet to be identified [16].

Co-infection and co-circulation of different HAdV types within the same species are necessary for the recombination process, which shuffles genome fragments within a species, but not between species [21]. According to Walsh et al., the genomes of HAdV-2, HAdV-6, and HAdV-57 are similar, with the only major differences occurring in the hexon and fibre regions [4]. This indicates that they have a shared ancestor and that HAdV-C6 may have been created through recombination within the hexon region of HAdV-C2, while HAdV-C57 may be a consequence of recombination within the fibre region of HAdV-C6. Despite the similarity between the hexon and fibre sequences of HAdV-C89 and the HAdV-C2 prototype sequence, as well as most of its genetic backbone, a novel penton base sequence has been identified in HAdV-C89 [4,5]. Given the close relationship between the parent strains C2, C6, and C89, TJ-Sewage-21110206 and TJ-ET165-2021, despite classification in two different recombinant patterns and having different isolation sources, likely have some connection in their evolutionary history.

Extensive research has strongly indicated that natural recombination of HAdV-C strains is vital for viral evolution and immune system avoidance [22]. Research has shown that the emergence of new HAdVs is due to the recombination of two or more viruses from the same species, and frequent coinfection probably encourages the natural recombination of HAdV-C species [6,15,23]. By characterising recombination events, indirectly determining which viruses are circulating at a specific location and time, this provides a better understanding of the viruses currently in circulation and the speed at which they have been replaced.

HAdV-C establishes long-term latent infections characterised by persistent intermittent excretion in nasopharyngeal secretions and faeces for months or even several years [24]. This may explain why many recombinants were isolated from sewage. Saliva samples may be used to identify a range of uncommon genotypes of HAdVs, which may rarely be detected in clinical human specimens. Compared with human surveillance techniques, such as testing and tracing infected individuals, sewage surveillance is a more reliable method for detecting early signs of community-level infections and disease outbreaks [25]. Evidence has shown that sewage surveillance may be used as an alert system for the emergence of HAdV, similar to poliovirus sewage surveillance that has been employed with the same goal [26]. This study confirms the value of viral recombinant analysis from sewage for public health surveillance.

## Figures and Tables

**Table 1:** Genome sizes and GC contents of 16 Tianjin strains.

Sequence	Genome Sizes	GC Contents (%)
TJ-ET165-2021	35,390	55.33
TJ-Sewage-210101	35,900	55.45
TJ-Sewage-210201	35,835	55.31
TJ-Sewage-210202	35,774	55.30
TJ-Sewage-210402	35,795	55.33
TJ-Sewage-210406	35,644	55.22
TJ-Sewage-21060601	35,765	55.22
TJ-Sewage-21110103	35,786	55.27
TJ-Sewage-21110104	35,761	55.18
TJ-Sewage-21110206	35,773	55.21
TJ-Sewage-21120204	35,815	55.27
TJ-ET149-2022	35,751	55.31
TJ-Sewage-22020104	35,772	55.24
TJ-Sewage-22020105	35,790	55.22
TJ-Sewage-22020201	35,774	55.23
TJ-Sewage-22030101	35,790	55.27

**Table 2:** The nucleotide identities between 16 Tianjin strains and HAdV-C representative strains.

Sequence	Nucleotide Identity (%)					
	HAdV-C1	HAdV-C2	HAdV-C5	HAdV-C6	HAdV-C57	HAdV-C89
TJ-ET165-2021	98.45	99.08	98.62	98.91	97.54	98.87
TJ-Sewage-210101	97.31	97.51	98.22	98.19	96.69	97.58
TJ-Sewage-210201	99.31	98.20	98.36	96.93	97.27	98.26
TJ-Sewage-210202	99.03	98.23	98.59	97.04	97.57	98.37
TJ-Sewage-210402	99.03	98.42	98.51	97.12	97.59	98.43
TJ-Sewage-210406	98.59	99.05	98.61	98.75	97.54	98.90
TJ-Sewage-21060601	98.44	98.53	99.00	98.58	98.63	98.70
TJ-Sewage-21110103	98.63	98.97	98.68	98.33	97.48	98.80
TJ-Sewage-21110104	98.47	98.53	98.99	98.62	98.68	98.71
TJ-Sewage-21110206	98.20	98.94	98.62	98.77	97.46	99.52
TJ-Sewage-21120204	98.55	99.07	98.62	98.85	97.52	98.85
TJ-ET149-2022	99.03	98.28	98.66	97.07	97.56	98.41
TJ-Sewage-22020104	98.55	99.03	98.57	98.74	97.53	98.82
TJ-Sewage-22020105	98.41	99.50	98.58	98.18	97.57	98.80
TJ-Sewage-22020201	98.19	99.68	98.86	98.60	97.44	98.94
TJ-Sewage-22030101	98.28	98.94	98.62	98.28	97.46	98.77

**Table 3:** The most closely identical strains in the GenBank and their nucleotide identity with 16 Tianjin strains.

Sequence	Most Closely Identical Strain in GenBank	Nucleotide Identity (%)
TJ-ET165-2021	ON054624	99.82
TJ-Sewage-210101	MK041227	99.41
TJ-Sewage-210201	MK041244	99.59
TJ-Sewage-210202	MT263140	99.77
TJ-Sewage-210402	MH183293	99.37
TJ-Sewage-210406	ON054624	99.90
TJ-Sewage-21060601	MK041234	99.36
TJ-Sewage-21110103	MF315029	99.89
TJ-Sewage-21110104	MK041242	99.29
TJ-Sewage-21110206	MH121114	99.63
TJ-Sewage-21120204	ON054624	99.82
TJ-ET149-2022	MT263140	99.78
TJ-Sewage-22020104	ON054624	99.88
TJ-Sewage-22020105	MH121084	99.85
TJ-Sewage-22020201	MZ151863	99.78
TJ-Sewage-22030101	MF315029	99.88

**Table 4:** Algorithms of the Recombination Detection Program (RDP) version 4 package used to predict the recombination events of 13 Tianjin strains.

Recombinant Strain	Parent Major/Minor	Recombinant Region in Alignment	Model (Average *p* Value)						
			RDP	GENECONV	BootScan	MaxChi	Chimaera	SiScan	3Seq
TJ-Sewage-210202	JX173083/ MF044052	28256–31639	3.604 × 10^−183^	7.272 × 10^−186^	1.532 × 10^−179^	5.392 × 10^−49^	2.865 × 10^−44^	1.688 × 10^−52^	4.419 × 10^−19^
TJ-Sewage-210402									
TJ-ET149-2022									
TJ-Sewage-210101	MK041227/ MK041234	100–6023	3.914 × 10^−66^	5.280 × 10^−61^	4.680 × 10^−53^	4.268 × 10^−19^	5.374 × 10^−17^	1.318 × 10^−18^	2.220 × 10^−15^
TJ-Sewage-21110103	KR699642/ JX173083	6740–18910	3.421 × 10^−46^	3.657 × 10^−18^	1.226 × 10^−15^	3.064 × 10^−12^	9.972 × 10^−14^	3.117 × 10^−12^	1.914 × 10^−5^
	KR699642/ KF268199	1361–6739	1.391 × 10^−43^	1.610 × 10^−5^	3.268 × 10^−29^	3.555 × 10^−10^	6.242 × 10^−4^	1.415 × 10^−7^	2.220 × 10^−15^
TJ-Sewage-22030101	KR699642/ JX173083	6762–18910	5.498 × 10^−46^	4.178 × 10^−19^	6.486 × 10^−15^	2.483 × 10^−12^	3.145 × 10^−04^	2.221 × 10^−12^	3.111 × 10^−05^
	KR699642/ KF268199	144–6206	9.919 × 10^−43^	1.592 × 10^−05^	1.700 × 10^−28^	4.405 × 10^−3^	1.203 × 10^−3^	2.814E × 10^−7^	2.220 × 10^−15^
TJ-Sewage-21110104	MK041244/MK041241	15439–36508	1.795 × 10^−52^	4.478 × 10^−18^	9.904 × 10^−25^	6.752 × 10^−13^	9.492 × 10^−18^	2.382 × 10^−121^	1.193 × 10^−67^
TJ-Sewage-21060601		15439–36227							
TJ-Sewage-210406	MF044052/ MK357714	10299–19306	1.125 × 10^−44^	2.795 × 10^−52^	3.560 × 10^−20^	1.174 × 10^−12^	1.465 × 10^−10^	2.858 × 10^−13^	8.715 × 10^−11^
TJ-Sewage-22020104		9473–19306							
TJ-Sewage-21120204		8450–19306							
TJ-Sewage-21110206	MH121114/ LC068716	11758–15320	1.321 × 10^−35^	2.689 × 10^−27^	8.306 × 10^−33^	1.169 × 10^−10^	8.263 × 10^−11^	2.595 × 10^−09^	3.330 × 10^−15^
TJ-ET165-2021	KR699642/ LC068716	12982–18546	1.754 × 10^−33^	7.011 × 10^−22^	1.141 × 10^−23^	3.703 × 10^−11^	5.590 × 10^−11^	——	3.330 × 10^−15^

## Data Availability

The datasets used and/or analysed during the current study are available from the corresponding author on reasonable request. All the Tianjin HAdV-C sequences obtained during this study were submitted in GenBank under accession numbers OQ834910-OQ834925.
